# Complement Factor H‐Based Therapeutics: A Comprehensive Overview

**DOI:** 10.1002/eji.70241

**Published:** 2026-07-22

**Authors:** Sebastiaan M. W. R. Hamers, Emmy J. Buter, Richard B. Pouw

**Affiliations:** ^1^ Sanquin Research Landsteiner Laboratory of the Amsterdam University Medical Centers University of Amsterdam Amsterdam the Netherlands; ^2^ Amsterdam Institute for Immunology and Infectious Diseases, Immunology Amsterdam the Netherlands; ^3^ Department of Hematology Amsterdam UMC Location VUmc Amsterdam the Netherlands

## Abstract

The complement system is a key component of innate immunity, modulating immunological processes to maintain proper homeostasis. However, dysregulation of the complement can lead to severe pathologies. Complement therapeutics strive to reduce and lower the risk of breakthrough complement activation, and the field has been growing steadily since the first complement‐regulating compound was introduced to the clinic in 2007, with many complement‐regulating strategies currently under development. This review focuses on therapeutic developments surrounding factor H (FH), which is a critical and native regulator of complement. FH is a complement inhibitor that controls the self‐amplifying alternative pathway (AP). It functions by destabilizing C3 and C5 AP convertases through decay‐accelerating activity, mainly via competition with factor B for binding to C3b. Additionally, FH acts as a cofactor for factor I, which cleaves C3b into iC3b. The classical and lectin pathways converge at the C3 level, triggering the AP self‐amplification loop. Independently, the AP is triggered by a tick‐over mechanism that is continuously activated. Both the amplification loop and the tick‐over mechanism of the AP are controlled by FH. Consequently, enhancing FH function in a dysregulated system represents a promising therapeutic approach. FH‐driven therapeutic strategies include replenishing FH via plasma‐derived or recombinantly produced full‐length FH. On top of that, protein‐engineered constructs that contain FH fragments both with and without targeting mechanisms are under development, as well as modulating moieties such as antibodies and peptides that bind and potentiate FH. In this review, we provide an extensive overview of these developments, discuss the underlying rationale of each strategy, and evaluate their status in the therapeutic pipeline.

## Graphical abstract

Complement factor H is a central regulator of the complement system and thus a target for therapeutic intervention. In this review, we provide an overview of past and current factor H‐based therapeutic concepts and modalities to treat complement‐mediated diseases.

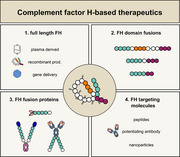

## 1 Introduction

### 1.1 Factor H Structure and Function

**FIGURE 1:**
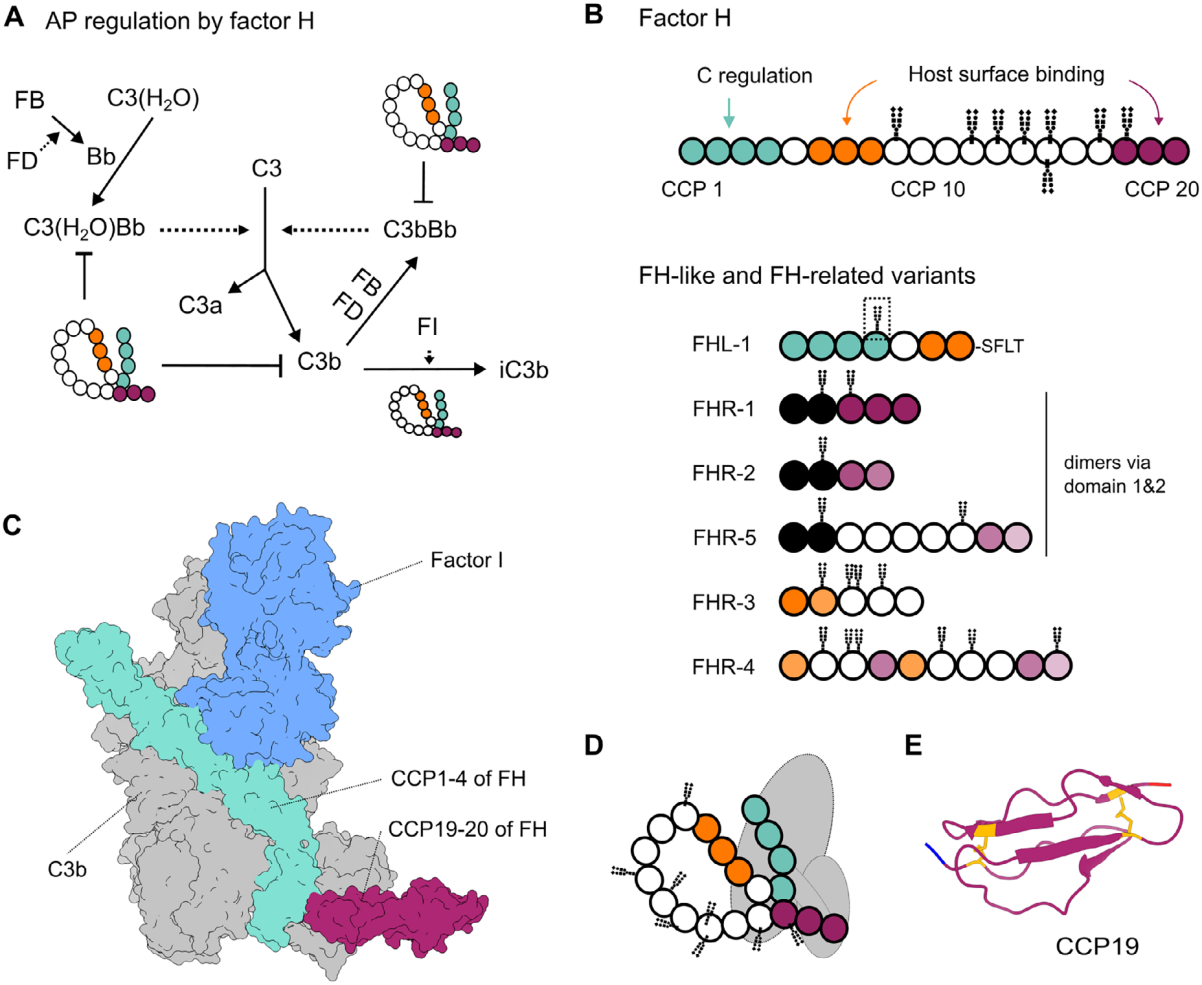
Structural aspects of factor H and the FH protein family. (A) FH regulates the self‐amplification loop of the alternative pathway of complement via decay‐accelerating activity or cofactor activity to FI. The loop can be initiated spontaneously via hydrolysis of C3, leading to C3(H_2_O) and subsequent binding of FB, which gets cleaved by FD. Alternatively, the C3 convertases of the classical or lectin pathway cleave C3 into C3b (only the cleaved C3b product displayed here for simplicity). Abbreviations: FD: factor D, FB: factor B, Bb: cleaved product of FB, C3: Complement component 3, C3a: cleaved product of C3, C3b: cleaved product of C3, iC3b: inactive C3b, C3(H_2_O): hydrolyzed product of C3, FI: factor I. (B) FH comprises 20 complement control protein domains (CCPs) and has nine potential glycosylation sites, of which 8 are usually glycosylated. The FH‐like (FHL) and FH‐related (FHR) protein families are structurally similar to FH and comprise a chain of CCP domains. The dimerizing CCP domains of FHR‐1, 2, and 5 are depicted in black, and the other functional domains that are homologous to FH are similarly color‐coded, with darker coloring indicating increased homology. Homology between the FHL/FHR and FH CCP domains lies between ∼25% and 100%. (C) The structure of the C3b‐FH‐FI complex (PBD: 5o35). This complex was elucidated with a truncated variant of FH (CCP1‐4,19‐20). CCP2 and 3 of FH interact with both C3b and FI and facilitate the cofactor activity of FH to FI. CCP19‐20 binds the C3d domain and allows interfacing with the surface of host cells. (D) The precise conformation of FH in complex with C3b is currently still unknown; this model, where FH is folded back on itself, is generally accepted. However, due to its length, FH could also span between two C3b proteins and form an inter‐C3b complex. (E) An enhanced view of a CCP domain to serve as an example. The general structure and size of CCPs are highly similar, and they contain two cysteine pairs (orange) that enhance structural stability. The N‐terminus (blue) and C‐terminus (red) are indicated.

Originally identified as the C3b binding protein beta 1H globulin, complement factor H (FH) is well known as a critical negative regulator of the complement system [1, 2]. The complement system is a branch of the immune system that comprises a set of over 50 proteins that are predominantly circulating in our blood. Complement facilitates inflammation, opsonization, and lysis of pathogenic cells and is propagated via proteolytic action [3, 4]. This powerful cascade, and particularly its spontaneously activating alternative pathway (AP), needs to be tightly controlled to prevent it from attacking and damaging host tissue (Figure 1A) [5].

Factor H is a glycosylated serum protein comprising 20 complement control protein (CCP, also known as Sushi or short consensus repeat) domains, which are linked in tandem (Figure 1B) [6]. CCPs are approximately 60 amino acids in size and contain two internal disulfide bonds (Figure 1E). These distinct domains are connected either directly or via a short flexible linker. CCPs are quite modular, and this has led to the development of protein‐engineered “mini‐FH” constructs and FH fusions, discussed later in this review.

Regulatory domains CCP1‐4 facilitate FH binding to C3b, leading to blocking of factor B binding to C3(H_2_O) or C3b as well as facilitating decay‐accelerating activity of C3bBb protein complexes. These C3 convertases are thus prevented from further self‐amplification. Next to the steric effect of FH binding to C3b, FH is a cofactor of factor I (FI), leading to C3b conversion to iC3b [7, 8]. CCP7, 19, and 20 bind glycosaminoglycans (GAGs) of the host cells, leading to specific recruitment of FH to host cell surfaces [9, 10, 11, 12]. This recruitment of FH to host cells is essential for proper AP regulation. Approximately 10% of the total mass of FH can be attributed to glycosylation. Glycosylation of FH occurs on CCP9, 12–15 (twice), 17, and 18, and most of these domains don't interact with FH ligands, although CCP18 has been found to bind pentraxins. Typically, eight out of the nine possible N‐glycosylation sites on FH are occupied, mostly by diantennary disialylated glycans [13]. It has been suggested that the heavily glycosylated center of FH acts as a hinge for FH to fold back on itself (Figure 1D). Furthermore, glycans on CCP17 and 18 have been implicated to drive FH dimerization and impact the efficacy of FH to protect host cells [14]. However, de‐glycosylated FH has been shown in vitro to have similar complement regulatory capacity as glycosylated FH [15].

Due to its inherent size and flexibility, capturing FH in the act for structural analysis is challenging. Therefore, no high‐resolution structure of full‐length FH exists. There are, however, several structures of CCP fragments available, also in complex with their respective ligands, which have been resolved via X‐ray crystallography [11, 16, 17, 18, 19]. In addition to these, a model of the C3b‐FH_ccp1‐4,19‐20_‐FI complex is available, which demonstrates the mode of action of FH cofactor activity of FI on C3b [20]. This model was built based on lower‐resolution cryogenic electron microscopy (CryoEM) structural data of the full protein complex. In the resulting model, high‐resolution X‐ray crystal structures were mapped to achieve a high‐resolution rendering of the complex. In this structure, the interface of C3b‐FH, FH‐FI, and C3b‐FI has been demonstrated simultaneously. Domains CCP2‐3 interact with FI on C3b, and the flexible serine protease domain of FI rigidifies and binds to C3b, leading to conversion of C3b to iC3b.

### 1.2 FH Protein Family

The FH protein family, consisting of FH, FH‐like (FHL‐1), and FH‐related‐1 to 5 (FHR‐1 and FHR‐5), fine‐tunes the AP response [21]. FHL‐1 and the FHRs are structurally similar to FH; their CCP domains display close likeness to FH and are ordered in the same chain‐like architecture (Figure 1B). The serum/plasma levels of the FH family of proteins have recently been thoroughly examined by our group, and the serum protein levels and accompanying molar ratios with respect to FH can be found in Table 1 [22]. These levels were also determined in plasma and were highly comparable except for FHL‐1. For FHL‐1, the plasma levels were approximately 15% higher compared with serum, likely a consequence of the coagulation step performed when obtaining serum.

**TABLE 1:** FH protein family serum levels.

	FH	FHL‐1	FHR‐1/1^a^	FHR‐1/2^a^	FHR‐2/2^a^	FHR‐3	FHR‐4	FHR‐5
Median (µg/mL)^b^	279.00	0.96	12.45	4.34	1.21	0.71	2.50	1.36
IQR (µg/mL)	227.30–335.00	0.81–1.16	7.37–15.05	3.04–5.41	0.71–1.71	0.44–1.02	1.32–3.59	1.15–1.62
Molar ratio compared with FH		84.04	15.31	30.44	141.10	131.70	106.60	88.92

^a^ 1/1, 1/2, and 2/2 indicate the homo‐ and heterodimers of FHR‐1 and FHR‐2.

^b^ Serum levels based on 201 healthy individuals, determined by ELISA [22].

FHL‐1 is the most similar to FH, comprising seven CCP domains that are 100% identical to FH CCP1‐7; only the last four amino acids at its C‐terminus differ. Interestingly, the glycosylation site at position N217, which is nonglycosylated in FH, is generally thought to be glycosylated in FHL‐1 [13, 23]. The five FHR proteins are encoded by the *CFH*‐*CFHR* locus on 1q31.3 and expressed in tandem. FHR‐1, 2, and 5 share highly similar CCP1‐2 domains at the N‐terminus that induce homodimerization of FHR‐1, 2, and 5 and heterodimers of FHR‐1 and FHR‐2 [24, 25, 26]. FHR‐3 and 4 share conserved C‐terminal CCP domains. While FHR function is not understood as well as FH or FHL‐1, FHRs likely function as antagonists for FH and FHL‐1 by competing in ligand binding. Further details on the biology of the FH protein family are not within the scope of this manuscript. The FH protein family has been extensively reviewed in recent years [27, 28, 29, 30].

### 1.3 FH and Disease

As outlined above, FH protects host cells and surfaces from complement. The relevance of FH is underscored by its association with various diseases, in which complement‐mediated damage of host cells occurs. We refer to other extensive reviews on the role of FH and the FH protein family in disease and only briefly discuss the main aspects here [21, 27, 28, 31, 32, 33].

The numerous associations of FH with various pathologies drive the development of FH‐based therapeutics. The pathologies can be related to mutations or polymorphisms in *CFH*, as well as FH autoantibodies, and may give rise to either altered protein function, changed expression levels, and/or FH:FHR ratios [21, 29, 34, 35]. Renal diseases such as atypical hemolytic uremic syndrome (aHUS), C3 glomerulopathy (C3G), and IgA nephropathy (IgAN) are associated with altered or decreased FH function, but are all relatively rare [36, 37, 38]. The most common FH‐associated disease is the ocular disease age‐related macular degeneration (AMD), which is the leading cause of blindness in developed countries, and due to its high prevalence, a large potential market [39, 40]. Next to these, FH has been implied other pathologies, including systemic lupus erythematosus (SLE), lupus nephritis (LN), rheumatic arthritis (RA), endometriosis, cancer, neurodegenerative disease, sickle cell disease, and atherosclerosis, all reviewed elsewhere [21, 28, 29, 35]. Of note, the most commonly used in vivo model in regard to FH‐based therapeutics, the *Cfh*−/− mouse model, presents with a C3G phenotype, which may develop retinal abnormalities at a later age [41, 42].

Not all pathologies related to FH are due to ineffective FH. Certain bacterial strains have evolved mechanisms to recruit FH via CCP19‐20, 6–7, or 8–11, thereby effectively evading complement‐mediated clearance [43, 44]. The importance of this escape mechanism for bacterial survival varies. In some species, this mechanism is crucial, such as *Neisseria meningitidis*, and the FH binding protein (FHbp) is even part of the vaccine against this pathogen [45, 46]. For *Streptococcus pyogenes*, one study found that FH‐binding M protein was not indispensable and possibly not even beneficial to virulence [47]. Notable examples of other FH‐binding bacteria are *Neisseria gonorrhoeae*, *Hemophilus influenzae*, *Streptococcus pneumoniae*, *Staphylococcus aureus*, *Candida albicans*, *Plasmodium falciparum*, and *Borrelia burgdorferi* [48]. This also exemplifies the importance of FH within the complement system and simultaneously raises the challenge to maintain the protective role of complement against pathogens while leveraging FH as a therapeutic. For instance, increased FH levels have been associated with increased risk for *N. meningitidis* or *S. pneumococcus* infections [49, 50].

Several of the diseases listed above are target indications for the FH‐based therapeutic concepts discussed here. However, diseases where FH is not directly implicated in pathology could potentially still benefit from FH‐driven therapeutic development. In principle, any disease that is characterized by complement overactivation or dysregulation is a candidate. This, for instance, includes paroxysmal nocturnal hemoglobinuria (PNH), the first indication for eculizumab. In PNH, hematopoietic cells lack surface complement regulators DAF, CD55, and CD59, making erythrocytes especially vulnerable to alternative pathway‐mediated lysis [51]. Inhibition of C5 with eculizumab or ravulizumab is often effective, but expensive [52, 53, 54]. Utilizing the natural AP‐regulating function of FH could provide a safer and possibly cheaper alternative to C5 inhibition, and since hemolysis assays are convenient tests of therapeutic function, PNH erythrocyte assays are often included in preclinical testing of FH therapeutics.

As outlined above, FH is a well‐studied complement regulator, playing a pivotal role in controlling the complement system. As such, it has always enjoyed an interest as a possible therapeutic modality or target and has served as inspiration for novel therapeutic concepts. This includes strategies to replenish or replace defective FH with plasma‐derived or recombinant preparations, engineering novel proteins out of selected FH CCPs, sometimes combined with other complement proteins, or selectively targeting FH at certain sites or for specific diseases to modulate or recruit its regulatory capacity.

## 2 Supplementing Factor H

### 2.1 Plasma‐Derived and Recombinant FH

Plasma exchange therapy is used for aHUS, ANCA‐associated vasculitis (AAV), and C3G patients [55, 56, 57, 58]. It effectively replenishes functional complement regulators like FH or washes out triggers (i.e., autoantibodies). It is an intensive therapy, requiring the supply of healthy plasma, which is time‐consuming for patients and not without risk of serious side effects like anaphylactic shock [55]. With FH being a relatively abundant protein in healthy plasma, it seems feasible to purify FH from human plasma and provide it as a plasma‐derived protein therapy. Several attempts and protocols have indeed been established to obtain functional plasma‐derived FH and develop it as a therapeutic (Table 2). Already in 2006, Laboratoire Francais du Fractionnement et des Biotechnologies (LFB) patented a method for preparing FH from cryoprecipitated human plasma, with a ∼28% yield and 91% purity [59]. It received orphan drug status in 2008 [59, 60]. Plasma‐derived FH was shown to be functional in *Cfh*−/− mice, and human clinical trials were planned to begin in 2011 [56, 61]. However, the orphan drug status was withdrawn on request of the sponsor in 2012, without further details given [60]. In the same period, both Octapharma and Baxalta Pharma filed patents describing methods to purify FH from fractions coming out of the Cohn fractionation procedure [62, 63]. The method described by Brandstätter et al. [63] yielded 14% plasma‐derived FH, with a 94% purity. Of note, a more recent protocol was also described using Cohn fraction III as starting material, achieving 92% purity and an improved yield of 39% [64]. Proof of concept was demonstrated with *Cfh*−/− mice, showing plasma‐derived human factor H reduces C3 deposition in the mouse glomeruli [64]. To date, there have been no clinical trials with plasma‐derived FH, and it remains uncertain whether plasma‐derived FH would indeed be a cost‐effective and successful treatment option.

**TABLE 2:** Full‐length FH productions.

Format	Name	Source	Status^a^	Company	Ref.
Plasma‐derived		Cryoprecipitated plasma	Withdrawn in 2012	LFB	[59, 60]
Plasma‐derived		Cohn fraction II+III	Patent active	Takeda Pharmaceuticals	[62]
Plasma‐derived		Cohn fractionation	Preclinical	Octapharma	[63]
Plasma‐derived		Cohn fraction III	Preclinical		[64]
Recombinant		*Spodoptera frugiperda* Sf9 cells	Preclinical		[65]
Recombinant		*Pichia pastoris*	Preclinical		[66]
Recombinant		*Cercopithecus aethiops* COS‐7 cell line	Preclinical		[67]
Recombinant		PER.C6 human cell line	Preclinical		[68]
Recombinant	GEM103	Unnamed mammalian cell line	Phase 2 ‐ terminated (NCT04246866, NCT04643886, NCT04684394)	Gemini Therapeutics^b^	[69, 70]
Recombinant, hum. glycans	CPV‐101	*Physcomitrium patens*, modified	Preclinical	Eleva GmbH	[71]
Recombinant, mod. glycans	CPV‐104	*Physcomitrium patens*, modified	Phase 1 ‐ ongoing (NCT07483827)	Eleva GmbH	[72, 73]

^a^ Last known status based on published work or other communications.

^b^ Gemini Therapeutics merged with Disc Medicine and no longer develops complement therapeutics [74].

More progress has been made with recombinant FH preparations (Table 2). To avoid confusion with respect to the following sections, in this section, we only discuss full‐length recombinant FH productions containing CCP1‐20. Recombinant protein production is thought to be a more scalable, economical, and customizable means of production and could alternatively be used to provide functional FH to patients. The first recombinant FH production was done in the *Spodoptera frugiperda* (Sf9) insect cell line, yielding 5 mg/L of full‐length, active FH [65]. However, the expensive medium and nonhuman glycosylation generally exclude Sf9 as a producer for commercial therapeutics [75]. In contrast, recombinant production using the yeast *Pichia pastoris* is highly scalable and commercially compatible, requiring inexpensive medium and, moreover, secreting high‐kDa proteins very efficiently [75]. With only codon‐optimization, ∼3–5 mg/L of functional human FH could be extracted from *P. pastoris* cultures [66]. Optimizing fermentation conditions and adding a gene for protein‐disulfide isomerase increased yield to up to 15 mg/L of murine FH [76]. In both cases, the nonmammalian glycosylation was enzymatically trimmed down, while retaining complement‐inhibiting functionality, in accordance with previous experiments [15, 66, 76]. For research purposes, FH has also been produced in COS‐7 (*Chlorocebus aethiops*; African green monkey) cells, more closely mimicking production by a human cell [67]. LFB additionally explored the human HEK239F and PER.C6 cell lines as producers of recombinant FH, with unknown yields [77]. In line with this, the biotech company Gemini Therapeutics used a mammalian cell line to produce recombinant FH (GEM103) [69]. GEM103 made it through phase 1 (NCT04246866) and phase 2a clinical trials in both dry (NCT04643886) and wet (NCT04684394) AMD, demonstrating its safety and tolerability, before being terminated in 2022 due to an apparent lack of efficacy [70, 78, 79].

The most active research line on this method, which is currently still being actively pursued, is undertaken by the company Eleva GmbH (Freiburg, Germany) with a surprising, alternative route for production. In 2011, Büttner‐Mainik et al. [80] first reported the production of active recombinant human FH in moss (*Physcomitrella patens*). The advantages of *P. patens* include precise genomic and glyco‐engineering, safety, lack of animal‐derived medium needs, great scalability, and batch‐to‐batch stability [81]. In 2017, the host was genetically modified to lose plant‐specific glycosylation enzymes, modifying recombinant FH to fit human glycosylation patterns (CPV‐101) [71]. In 2024, CPV‐104 debuted as a moss‐grown FH with modified glycans [72]. The addition of terminal sialic acids enhanced *Physcomitrella*‐produced FH to exhibit a half‐life comparable to native FH. CPV‐104 was functional *in vi*vo in *Cfh*−/− mice and cynomolgus monkeys, and ex vivo in plasma samples of aHUS, C3G, and PNH patients [72, 73]. Moreover, both CPV‐104 and CPV‐101 showed efficacy in an in vivo model of AMD [82]. As of 2025, CPV‐104 has received orphan drug status for C3G, and a phase 1 trial has been initiated (NCT07483827).

### 2.2 Gene Delivery

An alternative method of delivery to replenish FH is being explored: gene therapy. An important advantage of this strategy is the potential of single‐dose curation, while antibodies like eculizumab require intravenous injection every 1–2 weeks. Constitutive expression also overcomes loss of efficacy due to a short half‐life. To provide a longer‐lasting solution, researchers investigated FH gene therapies as early as 2015 [83]. Gene delivery through adeno‐associated virus vectors has been particularly explored for treating AMD, with both full‐length FH and FH fusion proteins (discussed below) [83, 84, 85]. Successful gene delivery and expression have been confirmed in vivo, and different injection sites have been explored, including subretinal, intravitreal, intraperitoneal, and intravenous. In addition, correcting *CFH* SNPs using CRISPR‐Cas9 is being investigated as an alternative approach to restore FH function [86].

## 3 Minimal‐FH and FH Fusion Proteins

### 3.1 Mini‐FH

Owing to the structurally defined CCP domains and their mapped individual roles within FH, the chain‐like organization of FH lends itself perfectly for tailor‐made, truncated variants. These truncated FH variants, or mini‐FHs, were designed to explore and perhaps improve FH function using a selection or combination of CCP domains across the FH backbone. The minimal‐FH protein designs published so far often omit CCP9‐17, which are not currently attributed with crucial functions (excluding a possible role in the folded‐back overall conformation of FH) and rather only include ligand binding domains of FH (Table 3) [35]. Production of these simplified protein constructs can be advantageous compared with full‐length FH, since size reduction and omission of glycosylation sites improve production yield as well as homogeneity. An additional theoretical advantage of a truncated FH is the avoidance of immune evasion by pathogens like *N. meningitidis*, by excluding domains that are exploited by bacterial FH‐binding proteins [87]. What's more, mini constructs could display special attributes not found in native FH, leading to functioning beyond baseline FH activity.

**TABLE 3:** Mini‐FH constructs.

Protein name	Domains	Source	Status^a^	Company	Ref.
Mini‐FH	1‐4, 19–20 (6 aa linker)	*S. frugiperda* Sf9 cells	Preclinical		[88]
AMY‐201	1‐4, 19–20 (G12 linker)	*P. pastoris*	Preclinical	Amyndas Pharmaceuticals	[89, 90]
Mini‐FH (extended)	1‐5, 18–20	Chinese hamster ovary (CHO) cells	Preclinical		[87]
FHΔ10‐15	1‐9, 16–20	*P. pastoris* (KM71H)	Preclinical		[91]
Midi‐FH	1‐4, 19–20, 1–4, 19–20	*P. pastoris* (KM71H)	Preclinical		[91]

^a^ Last known status based on published work or other communications.

**FIGURE 2:**
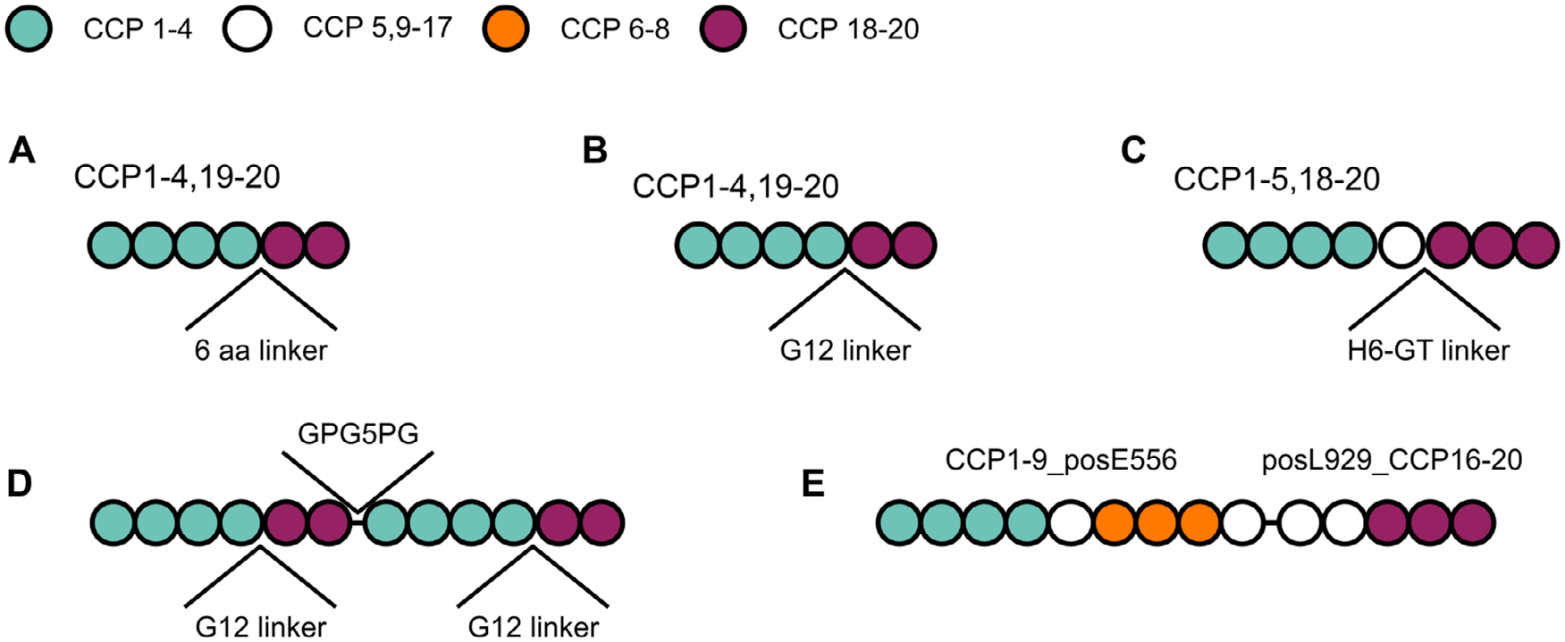
Truncated FH variants to create mini‐FH. The figure is color‐coded to the CCP domains of FH as in Figure 1. (A) Mini‐FH with short native linker [88]. (B) Mini‐FH with the polyglycine linker [89]. (C) Mini‐FH with the additional CCP domains 5 and 18 [87]. (D) Dimeric mini‐FH CCP1‐4,19‐20 poly G resulting in a midi‐FH variant [91]. (E) FHΔ10–15 is a truncated variant of FH that omits domain 10–15 [91].

Mini‐FH has been used as an encompassing term for several truncated FH variants; however, the exact CCP composition of a *mini‐FH* can differ (Figure 2). Nevertheless, there are certain principal assumptions. It is recognized that the main decay‐accelerating activity and cofactor activity of FH is driven by CCP1‐4, and all known mini‐FH constructs comprise these domains. Similarly, the host membrane targeting CCP19‐20 at the C‐terminus of FH is always included. Building on these initial insights, two groups independently developed rationally designed mini‐FH constructs (Figure 2A,B), which were developed to mimic native FH function in a simplified protein format with implications to develop toward a therapeutic [88, 89]. These constructs were highly similar, and the main difference between them was the linker connecting CCP4 to CCP19 (Figure 2A,B). While both were able to achieve similar functionality compared with full‐length FH in vitro, one of the constructs even surpassed FH function by tenfold in in vivo studies with a PNH model [89]. This polyG_12_‐linked CCP1‐4,19‐20 has more recently also proven effective in a mouse model of periodontitis [90].

Shortly after the introduction of CCP1‐4,19–20, an additional variant of a mini‐FH was described, which included domains CCP1‐5,18‐20 and displayed satisfactory results in vitro and in vivo with *Cfh*−/− mice via intraperitoneal injection. Unfortunately, the half‐life was less than 6 h, with obvious detriment to long‐term efficacy as compared with the 4–6 days for full‐length native FH [87].

Building on the initial lessons from mini‐FH variants, additional variants were evaluated that extend beyond the N‐terminal core of CCP1‐4. Native protein FHL‐1, which comprises CCP1‐7 of FH, and synthetic protein FHΔ10–15, which omits CCP10‐15, were produced to investigate this [91]. Omission of CCP10‐15 was hypothesized to positively influence the positioning of CCP19‐20 and thus enhance regulatory capacity on surfaces. These proteins displayed equal or improved regulatory activity compared with native FH. Additionally, to explore the effect of enhanced avidity, a CCP1‐4,19‐20 dimer was produced, which was fused head‐to‐tail via a short protein linker [91]. Both variants displayed superior protection of PNH erythrocytes compared with native FH and FHL‐1. Interestingly, cofactor activity of the truncated FHs, however, was not comparable to that of native FH, even though ligand‐binding affinities are similar [91].

With eculizumab, the bactericidal property of complement in serum is lost, leading to serious side effects in patients. Therefore, mini‐factor H with the G12 linker has been evaluated in a bactericidal assay [92]. At high concentrations of the mini‐FH spiked in human serum, the capacity of human serum to achieve bacterial killing was compromised. While the effect is not as drastic as with eculizumab, this should be evaluated over the course of therapeutic development. The authors point out that with lower mini‐FH concentrations, where PNH‐erythrocyte rescue still occurs, bacterial killing is nonsignificantly different from the normal serum control, so perhaps a dosing regimen should be adjusted accordingly [92].

While mini‐FH‐like constructs could be beneficial for renal pathologies, concerns have been raised about the theoretical effectiveness in retinal diseases, since CCP6‐7 have been reported to play an important role in AMD pathophysiology [93]. A notion supported by the fact that the AMD risk mutation Y402H is located on CCP7 [94]. Furthermore, FHL‐1 is deemed to be the principal regulator in Bruch's membrane and not FH [95]. In conclusion, proof‐of‐concepting mini‐FH has been promising; however, demonstration of prolonged efficacy in the preclinical setting has remained challenging. The most successful candidate of the mini‐FHs described here is the CCP1‐4, polyG_12_, CCP19‐20 fusion, which has been dubbed AMY‐201 and is under development with Amyndas Pharmaceuticals [90].

### 3.2 FH Fusion Proteins

Protein‐engineered constructs to drive complement regulation via FH are not limited to FH‐CCPs; numerous hybrids of complement control proteins, surface regulators, and antibody/antibody‐fragments have been produced (Table 4). Such fusions strive to utilize the most ideal properties derived from each part and facilitate protein targeting, half‐life extension, and enhanced avidity to arrive at an efficient complement controlling compound. In each of these studies, various protein‐engineered designs are compared; here, we discuss the most promising design of each study.

**TABLE 4:** FH fusion proteins.

Compound	FH part	Fusion protein	Function fusion protein	Status^a^	Company	Ref.
TT30	CCP1‐5	CR2, CCP1‐4	Target to tissues with iC3b/C3dg	Phase 1 (NCT01335165)	Alexion Pharmaceuticals	[96, 97, 98, 99]
Homodimeric mini‐FH (HDM‐FH)	CCP1‐5, CCP18‐20	FHR‐1, CCP1‐2	Dimerization	Preclinical		[100, 101]
MFHR1	CCP1‐4, CCP19‐20	FHR‐1, CCP1‐2	C5 convertase inhibition, dimerization	Preclinical		[102]
MFHR13	CCP1‐4, CCP13, CCP19‐20	FHR‐1, CCP1‐2	C5 convertase inhibition, dimerization	Preclinical		[103]
FH‐FHR5	CCP1‐5	FHR‐5, full length	Dimerization, renal targeting	Preclinical		[104]
B4‐scFv‐FH	CCP1‐5	Natural IgM fragment (Clone B4)	Target to tissues via annexin IV	Preclinical		[105]
C2‐scFv‐FH	CCP1‐5	Natural IgM fragment (Clone C2)	Target to tissues with PC phospholipids	Preclinical		[106]
Anti‐Annexin A2‐FH	CCP5‐20	Anti‐annexin A2 antibody	Target to site of complement activation	Preclinical		[107, 108]
IgG‐FH	CCP1‐5	IgG	Increase half‐life	Preclinical		[109]
Anti‐FP‐FH	CCP1‐5	Anti‐properdin antibody	Target to sites of complement activation	Preclinical		[109]
ADX‐097	CCP1‐5	Anti‐C3d antibody	Target to sites of complement activation	Phase 2 withdrawn (NCT06419205)	Q32bio	[110]
KP104	CCP1‐5	Anti‐C5 antibody	Inhibit C5, target to sites of complement activation	Phase 2 (NCT05476887, NCT05517980)	Kira Pharmaceuticals	[111, 112]
CG001	CCP1‐5	CRIg and Fc	CRIg: block C3b Fc: prolong half‐life	Preclinical		[113]
TRiFu	CCP19‐20	CD55, CCP1‐4 CR1, CCP15‐17	Inhibit all pathways	Preclinical	Quasar Therapeutics	[114]

^a^ Last known status based on published work or other communications.

**FIGURE 3:**
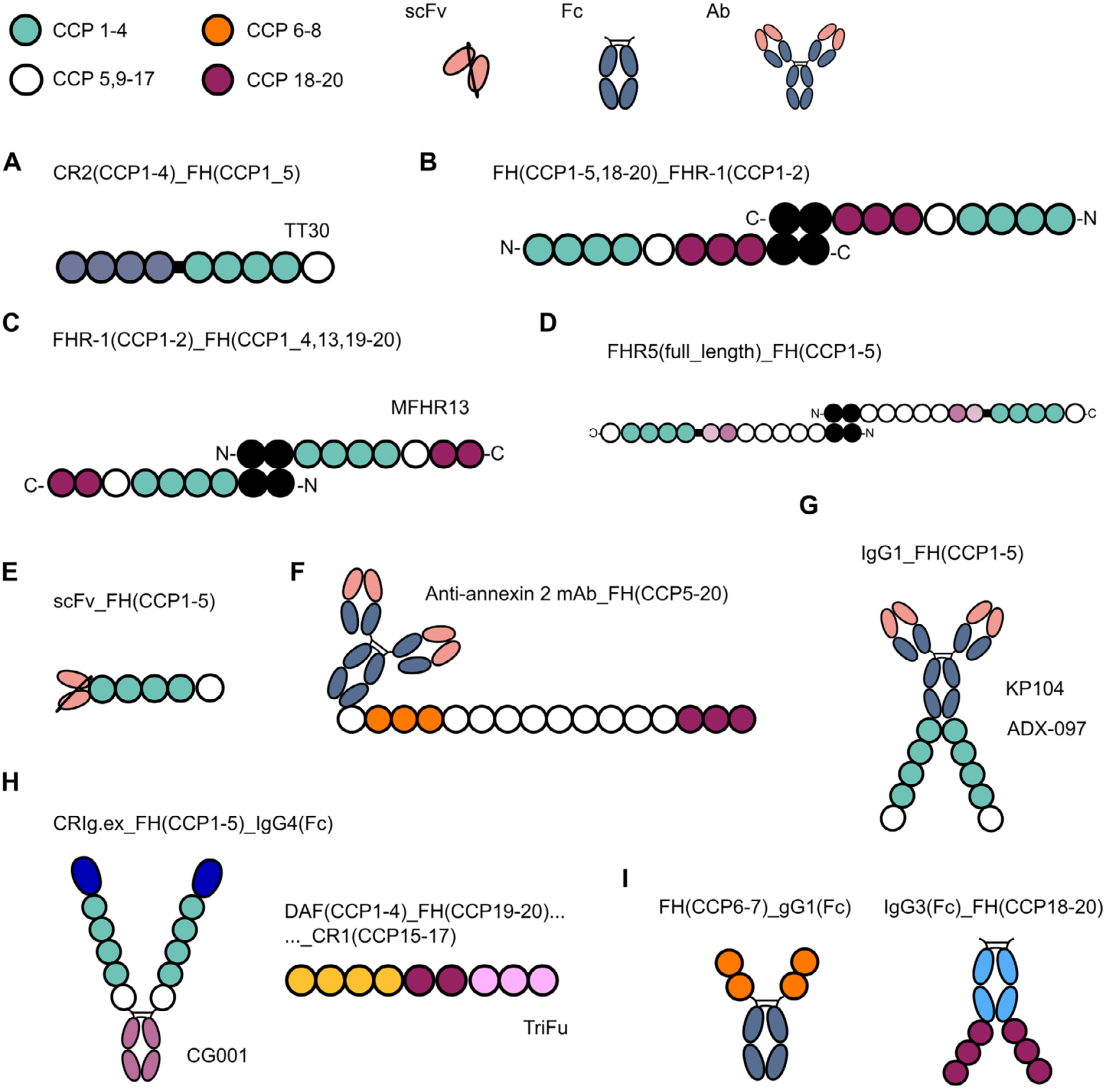
Structure of FH fusion proteins. The figure legend displays that FH domains in every fusion protein are color‐coded according to Figure 1, and antibody(‐domains) are shown with scFv: single chain variable domain; Fc: crystallizable fragment; and Ab: full‐length antibody. (A) In the TT30 fusion, the CR2 domains are annotated in dark grey [97]. (B) A mini‐FH fusion with the CCP1‐2 domains of FHR‐1 at the C‐term [100]. (C) MFHR13 is a mini‐FH fusion that contains CCP13 and the FHR‐1 CCP1‐2 domains at the N‐term [102]. (D) A full‐length FHR‐5 protein is fused with CCP1‐5 of FH. In (B–D), dimer‐forming FHR‐1/5 domains are shown in black [104]. (E) Natural antibody fusions scFv‐FH [105, 106]. (F) Full‐length antibody fusion to FHCCP5‐20 [107, 108]. (G) Monoclonal antibody fusion to FH_CCP1‐5. This generic design was used for ADX‐097 and KP105, as well as combined with a nonspecific and anti‐properdin IgG [109, 110, 112]. (H) Triple fusion proteins CG001 with the extracellular domain of CRIg (residues 19–137) depicted in dark blue and TriFu, which comprises DAFCCP1‐4 depicted in yellow, FHCCP19‐20, and CR1 depicted in pink [113, 114]. (I) The antibacterial fusion proteins. Design on the left utilizes CCP6‐7 at the N‐terminal section of the fusion, and the design on the right utilizes CCP18‐20 on the C‐terminal side of the fusion. The latter construct contains a mutation D1119G in the CCP domains that negates binding to self‐surfaces. Numbers mutation according to native FH [120, 121].

The most advanced FH‐fusion protein, now called TT30 (Alexion Pharmaceuticals), was initially described in 2008. In TT30 domains, CCP1‐4 of complement receptor 2 (CR2, CD21) are fused to FH_CCP1‐5 (Figure 3A) [96]. This was a second fusion protein of CR2, which had previously been combined with DAF‐ and CD59 [115]. The fusion enables dual regulation via CR2 that binds C3 fragments iC3b, C3d, and C3dg and thus targets sites of complement (over)activation. Here, fragment CCP1‐5 of FH can exert its complement‐regulating functions. This combinatorial approach has proven to be efficient as TT30 is reported to have a ∼150‐fold potency gain over FH [97]. Likewise, TT30 protects PNH erythrocytes from complement‐mediated hemolysis [98]. Furthermore, in vivo functions have been assessed in several disease models. In a mouse model for intestine ischemia/reperfusion injury, CR2CCP1‐4_FHCCP1‐5, but not FH, greatly reduced C3 deposition [96]. Then, in a choroidal neovascularization (CNV) mouse model, TT30 reduced CNV size and preserved retinal function [116, 117]. Even in healthy cynomolgus monkeys, TT30 inhibits AP activation [97]. The positive outcomes of these studies resulted in the start of a phase 1 clinical trial in PNH patients (NCT01335165). Although limited data are available for that study, TT30 was well‐tolerated and safe in 10 patients [118]. However, the short half‐life of TT30 (∼12–24 h) prohibits long‐term effects, which has led to termination of that research line [118]. While TT30, as an intravenous injection, was not further developed, its effectiveness displayed the potential of FH fragment fusions as semi‐natural modifiers of complement over‐activation, even in diseases with a molecular pathogenesis unrelated to FH. Currently, this fusion is still explored in ocular diseases, and studies are being conducted to develop gene and cell therapies, which wouldn't be hindered by the half‐life issues observed with the injections of recombinant protein [119].

The mini‐FH variants, as discussed above, displayed good potency but similarly unsatisfactory half‐life profiles [87, 100]. To improve this, several research lines have been developed. In one of these research lines, oligomerizing domains of FHR‐1 (CCP1‐2) are conjugated C‐terminally to FH CCP1‐5,18‐20, creating a homodimeric construct (Figure 3B) [100]. This dimer proved >10‐fold more effective than native FH in vitro and protected *Cfh−*/− mice significantly better than FH or mini‐FH. Additionally, the serum half‐life was enhanced fivefold [100]. However, due to the dynamic dimerization of human FHRs, a quick exchange with circulating FHRs in humans upon administration might affect the in vivo efficacy of this construct [25, 26]. It is noteworthy that in this study, a head‐to‐head comparison was made between C‐terminally and N‐terminally conjugated FHR‐1 domains 1–2, and it was concluded that the C‐fusion was overall better, although to a large extent highly comparable [100]. MFHR1 is a highly similar fusion from a different study, with the FHR‐1 domains indeed being on the N‐terminus of FHCCP1‐4,19‐20. MFHR1 used the original, shorter mini‐FH to conjugate to FHR‐1 domains [102]. Initially, MFHR1 did not outperform native FH in in vivo models, which was attributed to a lack of glycosylation sites and led to a successor molecule, named MFHR13 (Figure 3C). MFHR13 includes CCP13 and its glycosylation site, flexible linkers, and a positive electrostatic domain, which were hypothesized to be useful in enhancing surface recognition [103]. While cofactor activity did not exceed FH and decay acceleration, activity was similar between FH and MFHR13; MFHR13 regulates overall complement activity ∼37x more efficiently. What's more, a significantly increased heparin binding was indeed observed as compared with FH and MFHR1 [103]. The last dimerizing fusion that will be discussed has recently been published. Here, a full‐length FHR‐5 was fused to FHCCP1‐5 (Figure 3D) [104]. This was done to exploit the propensity of FHR‐5 to interact with the kidney complement. The fusion proved effective in murine models of C3G with either FH deficiency or human FH with an FHR‐5 mutation, indicating a potentially effective therapeutic approach [104].

Other modalities that target FH to sites of complement activation have also been explored. The targeting proteins in question, B4 and C2, are self‐reactive natural IgM antibodies (nAbs) [105, 119]. These are low‐affinity, polyreactive, and can recognize common microbes, but also damage‐associated patterns on self‐cells (DAMPs). In responding to DAMPs, nAbs tag apoptotic or damaged cells for removal and thus contribute to tissue homeostasis. B4 recognizes oxidatively stressed surfaces via a modified annexin IV, and C2 recognizes changed phospholipid epitopes and can thus target FH to sites of injury and AP activation [105, 122]. In the FH fusions, single‐chain variable fragments (scFv) were used and conjugated to CCP1‐5 (Figure 3E). B2‐scFv‐CCP1‐5 was tested in retinal injury mouse models, via a cell therapy delivery mode (encapsulated RPE cells), and was demonstrated to reduce CVN and complement activation [105]. C2‐scFv‐CCP1‐5 was explored in an acetaminophen liver injury model. C2‐scFv‐CCP1‐5 was found to reduce necrotic liver area, as well as decrease inflammation [106]. Although these experiments were conducted with mouse nAbs against murine epitopes, human IgM nAbs against AMD and oxidative stress epitopes have been identified [123].

IgG fusions have inherent therapeutic potential, which is enabled by high target affinity and specificity combined with relatively long half‐life profiles. Already in 2009, a patent was submitted that describes a FH‐fusion protein (CCP5‐20) with an anti‐annexin 2 mAb (Figure 3F) [107, 108]. Unfortunately, further information about this molecule is lacking. In a different study by Alexion Pharmaceuticals, two fusions of FH_CCP1‐5 to two mAbs have been investigated (Figure 3G). The antibodies used were an anti‐properdin and a nontargeting IgG [109]. Noticeably, no strong advantage of targeting with anti‐properdin over general IgG was found; the addition of either IgG to the CCP domains did provide the molecule with a significantly prolonged half‐life [109]. More recently, other promising experiments have been done with anti‐C5 (KP104, Kira Pharmaceuticals) and anti‐C3d (ADX‐097, Q32Bio) antibodies fused to FH_CCP1‐5 [110, 111, 112]. The aC5‐fusion has a dual function: (1) targeting FH to sites of complement activation to inhibit the alternative pathway, and (2) inhibiting the terminal complement pathway via the antibody itself. This has been a very successful combination as KP104 has since completed a phase 2 study (NCT05476887) in complement inhibitor‐naïve PNH patients [111]. While KP104 has a fairly complex safety profile with dual inhibitory action against complement, results so far have been promising, and a phase 2 study (NCT05517980) with KP104 in IgAN and C3G has been planned.

ADX‐097, the anti‐C3d mAb‐FH fusion protein of Q32Bio, provides targeting of CCP1‐5 to sites of complement activation [110]. ADX‐097 had promising preclinical data, and successful phase 1 and phase 2 clinical trials were expected to start in 2025 for diseases AAV, C3G, IgAN, and LN (NCT06419205) [124]. However, both are currently postponed indefinitely, while Q32Bio focuses on their alopecia areata drug [125].

From the dual we arrive at the triple fusion proteins, namely TriFu and CG001 (Figure 3I) [113, 114]. CG001 (ComGen, China) fuses the complement receptor of the immunoglobulin family (CRIg) to an Fc fragment and FH [113]. CG001 started as a CRIg‐FH fusion protein and has proven preclinical activity in MPGN rats, PNH erythrocytes, renal ischemia reperfusion injury mice, lupus nephritis mice, and myasthenia gravis rats [126, 127, 128, 129]. CRIg and FH bind C3b on different sites and synergistically inhibit alternative complement activation. The Fc fragment of an IgG4 was recently added to improve pharmacological properties and, in fact, improved binding affinity to C3b by ∼80‐fold (Figure 3I) [126]. (154). Following the promising preclinical results, a phase I trial in healthy volunteers was successfully finished, and a phase Ib trial is currently ongoing for PNH patients [113]. The next compound, TriFu (Quasar Therapeutics, Germany), combined FH, decay‐accelerating factor (DAF), and complement receptor 1 (CR1) (Figure 3I) [114]. The complex of these three endogenous complement inhibitors demonstrated a unique ability to inhibit all three complement pathways, as well as target sites of complement activation. While effective, TriFu also negated the bactericidal activity of serum, raising concerns about overall safety [114]. Nevertheless, bactericidal ability was diminished to an extent comparable to C5‐blockers; therefore, TriFu might provide an alternative to eculizumab and ravulizumab in PNH or diseases with broad complement overactivation.

### 3.3 FH Fusions to Combat Complement‐Resistant Bacterial Strains

The FH‐derived compounds discussed above have been designed to protect host cells from complement‐mediated damage. Alternatively, FH can even be used to direct complement activation toward a nonhost surface. Compounds exploiting this strategy are being generated to combat strains of bacteria that have evolved to hijack the complement regulatory ability of FH. Examples of these include Neisseria, Streptococcus, and Borrelia strains, as well as some fungi, protozoa, helminths, and viruses [44]. With antibiotic resistance ever‐increasing, novel anti‐infective therapeutics are urgently needed. Among the fifteen bacterial species that the WHO lists as priority threats, nine strains bind FH, highlighting a strong therapeutic necessity and opportunity [130]. Several strategies involving the FH‐pathogen interface have been explored, including sialic acid analogs; competitive inhibitors; factor H‐binding protein (FHBP) inhibitors; and vaccination with FHBP, which have been extensively summarized elsewhere [44].

The FH‐fusions to combat complement‐resistant bacteria drive complement activation by targeting the FH binding proteins on bacterial surfaces (Table 5). In these, bacteria‐binding FH domains CCP6‐7 or CCP18‐20 are linked to the Fc fragment of an IgG (Figure 3J). This relatively simple set‐up leads to a series of modulatory effects. (i) FH CCPs outcompete serum FH on the bacterial surface; (ii) via the covalent link, the Fc fragment is proximal to the bacterial surface; (iii) the Fc fragment activates the classical pathway and/or recruits phagocytes [120]. CCP6‐7/Fc has already been proven effective in *N. meningitidis*, *H. influenzae*, and *S. pyogenes* in infection models of transgenic rats and mice with human FH [120, 121, 131]. In these models, bacteria were efficiently cleared from blood and lung tissue, and enhancement of complement‐mediated killing of Gram‐negative bacteria and phagocyte‐mediated killing of Gram‐positive bacteria was observed.

**TABLE 5:** Antibacterial FH constructs.

FH part	Antibody	Targeted bacteria	Status^a^	Ref.
CCP6‐7	Human IgG1 Fc	*Haemophilus influenzae* *Neisseria meningitidis* *Streptococcus pyogenes*	Preclinical	[120, 121, 131]
CCP18‐20 (D1119G) C‐term	Human IgG1 or IgG3 Fc	*Neisseria gonorrhoeae*	Preclinical	[132, 133, 134]
CCP18‐20 (D1119G) N‐term	Human IgG1 Fc	*Staphylococcus aureus*	Preclinical	[135]

^a^ Last known status based on published work or other communications.

Multidrug‐resistant pathogens *N. gonorrhoeae* and *S. aureus* (MRSA) do not bind FH via CCP6‐7, but rather CCP18‐20 [132, 135]. However, since FH CCP18‐20 are also crucial in host‐recognition, mutations were explored to negate self‐binding, while retaining binding to the pathogen [132]. This need was met in the compound CCP18‐20(D1119G)/Fc, which significantly reduced infection burden in a mouse model infected with *N. gonorrhoeae* and did not induce hemolysis [132, 133]. The initial success of this fusion protein led to studies that further improved its efficacy. A systematically optimized protein construct implements a flexible linker between the CCP and Fc domains, inverted CCP positioning at the C‐terminus of the Fc, and applies a class switch from IgG1‐Fc toward IgG3‐Fc modification. These modifications led to a significant potency increase in the applied models [134]. FH18‐20(D1119G)/Fc was also effective for *S. aureus*, significantly increasing phagocytotic killing in serum [135].

## 4 Targeting FH

### 4.1 Nonantibody Proteins

At first glance, targeting a pivotal negative complement regulator does seem like a challenging approach, even more so when considering the various diseases associated with impaired FH function, due to genetic defect or autoantibodies, or the highly severe phenotype caused by FH deficiency [136]. Nevertheless, several compounds have been discovered and developed that target FH, with some even enhancing its function, while others aim to recruit FH to specific surfaces or biomaterials (Table 6).

**TABLE 6:** **Compounds targeting FH**.

Protein name	Class	Targeted domain	Effect	Status^a^	Company	Ref.
5C6	Peptide	CCP10‐14	Recruiting FH	Preclinical		[137, 138]
PspCN	Bacterial protein	CCP8‐10	Recruiting and enhancing FH	Phase 2 (NCT06070337)	Invizius	[139, 140]
Anti‐FH.07 (GEM307)	Antibody	CCP18	Enhancing FH	Preclinical	Gemini Therapeutics^b^	[141, 142]
Bispecific Ab	Antibody	CCP5 (OX24)	Recruiting FH	Preclinical		[143]
GT103	Antibody		Inhibit tumor‐derived FH	Phase 2 (NCT04314089, NCT05617313, NCT07017829)	Grid Therapeutics	[144, 145, 146, 147]
PolySIA‐NP	Nanoparticle		Activate FH	Preclinical	Aviceda Therapeutics	[148]

^a^ Last known status based on published work or other communications.

^b^ Gemini Therapeutics merged with Disc Medicine and no longer develops complement therapeutics [74].

The first molecule designed for targeting FH was 5C6, a cyclic peptide [137]. 5C6 was found using a phage‐display library and selected for binding to the middle domains of FH (CCPs 10–14), to refrain from interfering with immune regulatory functions. 5C6 binds FH with nanomolar affinity and thereby inhibits C3b deposition on the surface to which 5C6 is coated/bound. In addition, 5C6 is specific for FH and does not bind FHR proteins [138]. These properties may be particularly useful for safe use of biomaterials, including transplants, implants, nanoparticles, and hemodialysis membranes, which may be coated with 5C6 to prevent complement activation. 5C6 may even be used to protect cell surfaces [149]. 5C6 was found to bind FH via its cyclic core and C‐terminal region, making N‐terminal tethering to the biomaterial the preferred strategy [138].

While 5C6 was derived from a phage‐display library, the concept of binding FH to protect a surface against complement attack is actually a well‐known natural phenomenon, as many pathogens have evolved proteins capable of sequestering human FH to their surface. One such bacterial protein is *S. pneumoniae* surface protein C (PspC). The N‐terminal domain (PspCN) of this FH‐binding protein was found to bind especially tightly to FH, at CCP8‐10. Furthermore, PspCN forces FH into a previously uncharacterized conformation, exposing a cryptic binding site and doubling affinity for C3b [139]. Currently, PspCN is being explored as a protective measure for acute kidney injury (AKI) patients, with a phase 2b trial (NCT06070337) finished for H‐Guard (Invizius). As H‐Guard, PspCN is coated onto the dialysis membrane, a potentially immunogenic surface, recruiting the patient's FH during dialysis and thereby protecting against complement activation. Additionally, PspCN was found to enhance cofactor activity even of aHUS‐linked FH mutant proteins, making PspCN potentially capable of correcting functional FH deficiencies [140]. However, as PspCN is derived from a bacterial surface protein, immunogenicity issues and formation of anti‐drug antibodies are highly probable.

### 4.2 Antibodies

PspCN demonstrated that circulating FH can be functionally enhanced. Coincidentally, a monoclonal antibody was discovered around the same time with an identical functional effect on FH; anti‐FH.07 [141]. This mouse‐derived anti‐human FH antibody binds FH at a different domain (CCP18) compared with PspCN (CCP8‐10), but similarly increases the binding affinity of FH to C3b. This enhancement resulted in decreased C3b deposition on surfaces that are naturally able to bind human FH, like sheep red blood cells and human endothelial cells. Similar to PspCN, anti‐FH.07 could also restore the function of the mutated FH found in aHUS [142]. Importantly, anti‐FH.07 did not confer FH‐mediated complement regulation to the Gram‐negative bacteria *E. coli* and *N. meningitidis* [141]. This makes FH enhancement a unique approach as a potential complement therapeutic possibly without any increased risk of infections, in great contrast to the current therapeutics. Anti‐FH.07 was under development by Gemini Therapeutics as GEM307, with additional FH‐enhancing antibodies patented, but the program was discontinued after a merger of Gemini Therapeutics with Disc Medicine in 2022 [74].

Several other FH‐targeting mAbs have been developed with the aim of preventing systemic complement inhibition and increasing targeting to sites of complement overactivation. These were designed as bispecifics for FH and (auto)antigens specific to auto‐immune disease [143]. The autoantigens include gangliosides, targets in Guillain–Barré syndrome; the acetylcholine receptor/MUSK, against which autoantibodies form in myasthenia gravis; PR3 and MPO, targets in AAV; collagen, in arthritis; and more [150]. The combination of anti‐FH and anti‐disease‐specific target ensures that FH is recruited to sites of local complement overactivation, as supported by the bsAbs’ effective protection of antigen‐positive liposomes, leukocytes, and erythrocytes from complement‐mediated killing [143].

Contrary to the previous compounds, the next monoclonal does not target FH to enhance its complement‐inhibiting function but rather aims to block FH and increase complement‐mediated killing. GT103 is a fully human IgG3 that binds and inhibits FH in non–small cell lung cancer (NSCLC). Several cancers are known to produce and bind FH on their surface, possibly to evade complement‐mediated lysis [151, 152]. FH expression in breast cancer cells has been linked to an immunosuppressive environment [153]. In addition, auto‐antibodies against FH were found in some NSCLC patients and correlated to favorable outcomes (early stage disease and increased time to recurrence) [154]. Taking advantage of this finding, researchers produced recombinant anti‐FH antibodies from patients’ B cells [144]. GT103 was reported to bind a tumor‐specific conformational epitope on FH and cause enhanced complement‐dependent cytotoxicity and antibody‐dependent cellular phagocytosis of tumor cell lines, as well as inhibiting tumor growth and metastasis in vivo [144]. Importantly, GT103 was also capable of converting an immunosuppressive tumor microenvironment (TME) to a pro‐inflammatory one [146]. GT103 binds solely to lung tumor‐associated FH, while avoiding serum FH and other tissues, as evidenced by crystallography of a cryptic GT103‐binding site and immunofluorescence of human ex vivo tissue showing clear staining of tumors, but not of normal human lung tissue [146]. A single‐arm phase 1b study (NCT04314089) has been conducted with 31 refractory NSCLC patients, reporting the safety and tolerability of GT103 [147]. There was one event of grade 3 acute kidney injury, which is interesting given the predilection of FH‐deficiency diseases to the kidney. In general, however, GT103 seems safe, and phase 2 trials have started, assessing the effect of GT103 in combination with pembrolizumab in refractory NSCLC (NCT05617313) and STK11 mutant NSCLC (NCT07017829) [147]. While the specificity of GT103 to tumor‐bound FH seems quite remarkable, the current results support this observation, and as GT103 is indicated for advanced and refractory cancers, possible adverse effects are more justifiable, as they are with chemo‐ or radiation therapy.

### 4.3 Other

A new non‐mAb, nonpeptide approach to FH‐targeting is described by Peterson and colleagues (Aviceda, USA) [148]. Based on the natural ability of FH to bind to polyanionic molecules on the surface of self‐cells, this molecule is a poly‐sialic acid nanoparticle (PolySia‐NP). PolySia‐NP increased FH binding affinity for C3b; inhibited hemolysis of rabbit erythrocytes; decreased C3b deposition; markedly reduced C3 concentrations, and proved effective in an AMD mouse model [148]. It has a second function, unrelated to factor H, in inhibiting macrophage‐mediated inflammation through Siglecs [155]. This two‐pronged mechanism might work synergistically to reduce immune activation. A molecule targeting FH via domain 7 could be challenging in AMD, since ∼50% of AMD cases are associated with a Y420H polymorphism on domain 7, which reduces the ability of FH to bind to sialic acids [94]. However, PolySia‐NP seems able to bind similarly to Y402H FH and wild‐type FH. Potentiating factor H via a PolySialic acid nanoparticle is an exciting new idea. However, the question remains if stronger binding to C3b can be a disadvantage, since it might limit the re‐usability and thereby the activity of FH.

## 5 Translational Considerations

As with any experimental therapy, translation toward clinical use entails other considerations besides efficacy. Especially regarding complement inhibitors, safety concerns related to infection risks have always been a major hurdle, which for the current therapeutics has resulted in black‐box warnings, requirement of prior vaccinations and close monitoring of treated patients, including use of prophylactic antibiotics. As FH is an endogenous complement regulator, its use may be considered safer due to its inherent host specificity. This, in theory, would allow the complement system to exert its primary function in clearing pathogens, while protecting host cells from complement‐mediated damage.

However, increasing the FH concentration through replenishment with either plasma‐derived or a recombinant preparation does need to consider possible effects on infectious risks in the long term. Several studies have linked endogenous FH levels to bacterial survival in plasma and risk of infectious diseases. In vitro experiments with *Streptococcus pneumoniae* demonstrated that relatively small differences in FH concentration already translate into altered bacterial survival [49]. While this was shown in an isolated system without other components of the immune system present, in vivo FH levels have been linked to pneumococcal meningitis, meningococcal disease, and malaria [50, 156, 157]. Likely, a similar risk arises when using FH constructs or fusion proteins. As indicated before, Mini‐FH was shown to confer complement regulation to *E. coli*, and thus may still come with an increased infection risk in vivo [92]. This may be avoided in more targeted approaches (i.e., targeting tumor‐derived FH) or specialized/localized use (i.e., coated to biomaterials) of FH‐based therapeutics, but assessing effects on bacterial clearance remains an important step in preclinical development.

Another critical aspect of therapeutics is pharmacokinetics (PK). In vivo efficacy can only be achieved if sufficient drug levels can be maintained. Unfortunately, little is known about the in vivo clearance and half‐life of FH and FH‐derived proteins. Initial studies with FH (β1H) in 1979 demonstrated a half‐life of ∼3.2 days in healthy individuals [158]. In a more recent study, in which one complete FH‐deficient patient suffering from aHUS was successfully treated with plasmapheresis, a half‐life of ∼6 days for FH after transfusion was reported [159]. It is unclear whether this half‐life was affected by disease status, although others have shown in mice that clearance rates seem to be affected by the presence of C3/C3b, based on prolonged clearance rates in mice with combined *Cfh* and *C3* deficiency compared with *Cfh*−/− mice [61]. Plasma‐derived FH preparations are expected to show a similar half‐life, whereas production of recombinant preparations will need to take into account posttranslational modifications like glycosylation to improve half‐life. Only a few studies address this concern. For instance, Tschongov et al. [72] reported a half‐life for plasma‐derived human FH of ∼5.35 h, and ∼2.7 h for their moss‐derived recombinant FH with optimized human glycosylation in wild‐type mice. In nonhuman primates, the recombinant FH (CPV‐104) showed a half‐life of ∼50 h, but it remains unclear how this correlates with human clearance rates [72]. Antibodies that target FH may rely on well‐known antibody pharmacokinetics and strategies to improve this, and FH constructs that include immunoglobulin domains similarly show improved PK [109]. However, target‐mediated clearance may still pose a significant challenge, as little is known about how FH may be cleared and how this may be affected by a bound antibody. Results of the ongoing clinical trials will hopefully shed more light on this.

## 6 Concluding Remarks

As the central negative regulator of the AP, FH remains a target of high interest in the current age of complement therapeutics. The well‐characterized function of FH and its established involvement in a wide range of (complement‐mediated) diseases have sparked numerous attempts to either replenish FH, use its functional domains, or target it otherwise to therapeutically regulate undesired complement activation. These diverse AP‐regulating strategies via FH proteins and domains reflect that understanding the mechanism of action of FH enables creative protein engineering. While stimulating, the diversity of these approaches makes for a difficult head‐to‐head comparison. This is particularly difficult when trying to predict clinical success. The functional parameters of these compounds have been rigorously investigated in in vitro and in vivo models. However, clinical feasibility hinges on parameters such as ease of production, compound stability in therapeutic formulations, PK in humans, and similarity to previous therapeutic compounds. The evaluation of such parameters will become available as research progresses.

While initial attempts at developing a plasma‐derived FH preparation and other recombinant full‐length FH programs ceased, renewed efforts by Eleva with moss‐derived FH may finally prove FH as a suitable protein for replenishment therapy. Their FH production using moss seems to have tackled at least one important challenge in the scalability of a recombinant FH therapeutic product, and results from their clinical trials are eagerly awaited. What's more, recombinant constructs based on the functional CCPs of FH continue to be explored and developed, more often now in combination with CCPs from other complement regulators to create novel modalities that add new, synergistic functions to FH domains. In this regard, the antibody fusions KP104 and ADX‐097 with CCP1‐5 of FH have performed particularly well and are moving through the clinical trial pipeline. The inherent properties of antibodies with regard to target specificity and, in particular, their half‐life profile are important drivers to generate a successful compound. This has become clear when comparing these fusions to other candidates such as TT30, which was highly promising in both in vivo and in vitro studies, but lacked longitudinal efficacy.

Besides the replenishing and fusions of FH, molecular targeting of FH with antibodies, proteins, or peptides is being explored. Interestingly, the most advanced compound in the category is the GT103 antibody that targets a tumor‐cell‐induced structural version of FH to allow for complement activation, rather than inhibition. This highlights the importance of context in FH disease‐related biology.

As the complement therapeutics field is developing, head‐to‐head comparisons between different lead compounds are increasingly important, but rarely done. For FH‐based compounds, as described in the review, the well‐characterized *Cfh−*/− mouse model is commonly used across studies and offers some indirect means to compare compounds [61, 64, 72, 101, 102]. Across these studies, a common quick effect on restoring C3 levels was found within 24 h, and the most optimal reduction in C3 deposition within the glomeruli was observed within 4–5 days posttreatment with full‐length FH preparations, either recombinant or plasma‐derived [61, 64, 72, 73]. In contrast, the FH‐based constructs HDM‐FH and MFHR1 appeared less effective in this *Cfh−*/− model compared with full‐length human FH [101, 102]. This shows that the *Cfh−*/− model, or similar models, could serve as a tool to differentiate between FH‐based compounds and therapeutic strategies.

The field of complement therapeutics has notably expanded over the past decade, with Sutimlimab, Ravulizumab, Pegcetacoplan, Avacopan, Iptacopan, Danicopan, and Crovalimab targeting C1s, C5, C3/C3b, C5aR1, factor B, factor D, and C5, respectively [160]. With these compounds, it is now possible to target the classical and AP as well as have alternative options for terminal pathway targeting instead of Eculizumab. With increased understanding of complement‐mediated disease and more tools available in the therapeutic toolbox, patients’ outcomes are expected to improve. While this expansion of treatment options is impressive, their efficacy in various pathologies is variable. Thus, there is a clinical need for additional complement therapeutics, in particular therapeutics that also negate the strong side‐effects and perhaps offer more targeted/local approaches. FH‐based therapeutics hold the potential to fill this gap, and while clinical trials are underway and extensive preclinical efforts have been made so far, a FH‐based therapeutic is not currently in the clinic. Nevertheless, all the combined efforts and diverse strategies that have been described here provide a clear indication that an FH‐based approach could deliver a viable therapeutic.

## Author Contributions

S.M.W.R.H., E.J.B., and R.B.P. conceived and drafted the manuscript. S.M.W.R.H. created the figures. S.M.W.R.H. and R.B.P. revised the manuscript content and format.

## Funding

The authors have nothing to report.

## Conflicts of Interest

R.B.P. is a co‐inventor on patents and patent applications describing the therapeutic use of anti‐FH antibodies and has been supported by Gemini Therapeutics. The remaining authors declare no conflicts of interest.
